# Concurrent Initialization for Bearing-Only SLAM

**DOI:** 10.3390/s100301511

**Published:** 2010-03-01

**Authors:** Rodrigo Munguía, Antoni Grau

**Affiliations:** Automatic Control Department, Technical University of Catalonia, c/ Pau Gargallo, 5 E-08028 Barcelona, Spain; E-Mail: rodrigo.munguia@upc.edu

**Keywords:** bearing-sensor, SLAM, robotics

## Abstract

Simultaneous Localization and Mapping (SLAM) is perhaps the most fundamental problem to solve in robotics in order to build truly autonomous mobile robots. The sensors have a large impact on the algorithm used for SLAM. Early SLAM approaches focused on the use of range sensors as sonar rings or lasers. However, cameras have become more and more used, because they yield a lot of information and are well adapted for embedded systems: they are light, cheap and power saving. Unlike range sensors which provide range and angular information, a camera is a projective sensor which measures the bearing of images features. Therefore depth information (range) cannot be obtained in a single step. This fact has propitiated the emergence of a new family of SLAM algorithms: the Bearing-Only SLAM methods, which mainly rely in especial techniques for features system-initialization in order to enable the use of bearing sensors (as cameras) in SLAM systems. In this work a novel and robust method, called *Concurrent Initialization*, is presented which is inspired by having the complementary advantages of the Undelayed and Delayed methods that represent the most common approaches for addressing the problem. The key is to use concurrently two kinds of feature representations for both undelayed and delayed stages of the estimation. The simulations results show that the proposed method surpasses the performance of previous schemes.

## 1. Introduction

Simultaneous Localization and Mapping (SLAM) is perhaps the most fundamental problem to solve in robotics in order to build truly autonomous mobile robots. SLAM treats of the way how a mobile robot can operate in an *a priori* unknown environment using only onboard sensors to simultaneously building a map of its surroundings which uses to track its position.

The robot’s sensors have a large impact on the algorithm used for SLAM. Early SLAM approaches focused on the use of range sensors as sonar rings or lasers e.g., [1]. Nevertheless there are some disadvantages with the use of range sensors in SLAM: correspondence or data association is difficult; they are expensive and some of them are limited to 2D maps and computational overhead due to large number of features (see [2,3] for a complete review).

The aforementioned issues have propitiated that recent work moves towards the use of cameras as the primary sensing modality. Cameras have become more and more interesting for the robotic research community, because they yield a lot of information for data association, although this problem remains latent. Cameras are well adapted for embedded systems: they are light, cheap and power saving. Using vision, a robot can localize itself using common objects as landmarks.

On the other hand, while range sensors (*i.e.*, laser) provide range and angular information, a camera is a projective sensor which measures the bearing of images features. Therefore depth information (range) cannot be obtained in a single frame. This fact has propitiated the emergence of a new family of SLAM methods: The Bearing-Only SLAM methods, which mainly rely in especial techniques for features system-initialization in order to enable the use of bearing sensors (as cameras) in SLAM systems.

In this context, a camera connected to a computer becomes a position sensor which could be applied to different fields such as robotics (motion estimation for generally moving robots humanoids), wearable robotics (motion estimation for camera equipped devices worn by humans), tele-presence (head motion estimation using an outward-looking camera), or television (camera motion estimation for live augmented reality) [4].

Usually the Bearing-Only SLAM has been associated with vision-based SLAM systems, possibly because cameras are by far the most popular bearing sensor used in robotics. In that sense, the use of alternative bearing sensors (*i.e.*, auditory sensing) for performing SLAM has been much less explored. Nevertheless in an authors’ previous work [5], a Sound-Based SLAM system is proposed where sound sources are used as map features and thus showing the viability on the inclusion of the hearing sense in SLAM and the use of alternative bearing sensors.

In recent years several important improvements and variants to this kind of methods have appeared [6,7]. Also different schemes for increasing the number of features managed into the map have appeared [8]. Nevertheless the initialization process of new features is still the most important problem for addressing in Bearing-Only SLAM in order to improve the robustness.

In this work a novel and robust method called *Concurrent Initialization* is presented which is inspired by having the complementary advantages of the Undelayed and Delayed methods, which represent the most common approaches for addressing the problem of initializing new features in bearing-only SLAM.

## 2. Related Work

Bearing-Only SLAM has received most attention in the current decade. Therefore many of the related approaches are actually very recent. In [9] Deans proposes a combination of a global optimization BA, (Bundle Adjustment) for feature initialization and Kalman Filter for state estimation. In this method, due to the limitation on the baseline on which features can be initialized and depending on the camera motion and the landmark location, some features cannot be initialized. Strelow proposes in [10] a similar method but mixing camera and inertial sensors measurements in an Iterated Extended Kalman Filter (IEKF). In [11] Bailey proposes a variant of constrained initialization for bearing-only SLAM, where past vehicle pose estimates are retained in the SLAM state so that feature initialization can be deferred until their estimates become well-conditioned. In that sense the past poses of the robot are stacked in the map, together with associated measures, until base-line is sufficient to permit a Gaussian initialization. The criteria used for determining whether the estimation is well-conditioned (Gaussian) is the Kullback distance. The complexity of the proposed sampling method to evaluate this distance is very high.

Also there are some works that use other estimation techniques (apart to the EKF) in Bearing-Only SLAM like the Particle Filters (PF) based methods. In a series of papers, Kwok uses Particle Filters: in [12] variations to standard PF are proposed to remedy the sample impoverishment problem in bearing-only SLAM. In [13] initial state of features is approximated using a sum of Gaussians, which defines a set of hypothesis for the position of the landmark, and includes all the features inside the map from the beginning. On successive observations, sequential radio test (SRT) based on likelihoods is used to prune bad hypothesis. The way these hypotheses are initialized is not detailed, and convergence and consistency issues are not discussed. In [14] Kwok extends the algorithm using a Gaussian Sum Filter, with an approach similar to the proposed in [15] for bearing-only tracking. This method is perhaps the first undelayed feature initialization method. The main drawback of this approach is that the number of required filters can grow exponentially, and therefore computational load grows exponentially with the number of landmarks.

Some of the most notably advances on Bearing-Only SLAM have been presented by Davison [4,16], who shows the feasibility of real-time SLAM with a single camera, using the well-established EKF estimation framework. In this work a Bayesian partial-initialization scheme for incorporating new landmarks are proposed where a separate Particle Filter is used for estimating the feature depth which is not correlated with the rest of the map. In that sense it maintains a set of depth hypotheses uniformly distributed along the viewing ray of a new landmark, with a particle filter in one dimension. Each new observation is used to update the distribution of possible depths, until the variance range is small enough to consider a Gaussian estimation, in this point the estimation is added to the map as a three-dimensional entity. Until this initialization occurs, the ray estimation is maintained in the system`s single Extended Kalman Filter. A drawback of this approach is that the initial distribution of particles has to cover all possible depth values for a landmark, this fact makes it difficult to use when the number of detected features is large or when there are far features in the scene. As a result, its application in large environments is not straightforward, as it would require a huge number of particles.

Jensfelt in [17] presents a method where the idea is to let the SLAM estimation lag behind N frames and using these N frames to determine which points are good landmarks and find an estimate of their 3D location. Mainly the focus in this work is on the management of the features to achieve real-time performance in extraction, matching and loop detection.

In [18] Sola presents a method based on Federate Kalman Filtering technique. With initial Probability Distribution Function (PDF) for the features, a geometric sum of Gaussians is defined. The method is an approximation of the Gaussian Sum Filter (GSF), which was used in [14], that permits undelayed initialization with simply an additive growth of the problem size. A drawback of this approach is that it does not cope with features at very large depths. In [19] Sola presents a similar method to the presented one in [18] but features are initialized with a delayed method.

In [20] a FastSLAM [21] based approach is proposed by Eade. Here the pose of the robot is represented by particles and a set of Kalman Filters refines the estimation of the features. When the inverse depth collapses, the feature is converted to a fully initialized standard Euclidean representation. This approach for features initialization seems appropriate within a FastSLAM implementation, but it lacks for a more general framework.

Montiel *et al.* in [22] presented a method, where the transition from partially to fully initialized features does not need to be explicitly tackled, making it suitable for direct use in EKF framework for sparse mapping. In this approach the features are initialized in the first frame observed with an initial fixed inverse depth and uncertainty, determined heuristically to cover range from nearby to infinity, therefore distant points can be coded. Due to the clarity and scalability, this approach is a good option to be implemented.

On the other hand, experiments show that initial fixed parameters can affect the robustness of the method, especially when an initial metric reference is used in order to recover/set the scale of the map. This fact motivated the authors to develop in [23] a delayed version of the above method. In this case, initial depth and uncertainty of each feature are dynamically estimated prior to add this new landmark in the stochastic map. The works [22] and [23] are analyzed in the next sections. Table 1 shows a summary of the above methods.

## 3. Problem Statement

### 3.1. Sensor motion model

Let us consider a bearing sensor, with a limited field of view, moving freely in 2DOF. The sensor state x̂*_v_* is defined by:

(1) $${\hat{x}}_{v}={\left[x_{v},y_{v},\theta_{v},v_{x},v_{y},v_{\theta }\right]}^{T}$$

where [*x_v_, y_v_, θ_v_*] represents the center position and orientation of the sensor and [*v_x_, v_y_, v_θ_*] denoting linear and angular velocity.

At every step it is assumed an unknown linear and angular acceleration with zero mean and known covariance Gaussian processes, *a*^W^ and α^W^, producing an impulse of linear and angular velocity:

(2) $$n=\left[\begin{array}{c} V_{x}^{W}\\ V_{y}^{W}\\ V_{\theta }^{W} \end{array}\right]=\left[\begin{array}{c} a_{x}^{W}\Delta t\\ a_{y}^{W}\Delta t\\ a_{\theta }^{W}\Delta t \end{array}\right]$$

The sensor motion prediction model is:

(3) $$f_{v}=\left[\begin{array}{c} x_{v(k+1)}\\ y_{v(k+1)}\\ \theta_{v(k+1)}\\ \begin{array}{l} v_{x(k+1)}\\ v_{y(k+1)}\\ v_{\theta (k+1)} \end{array} \end{array}\right]=\left[\begin{array}{c} x_{v(k)}+\left(v_{x(k)}+V_{x}^{W}\right)\Delta t\\ y_{v(k)}+\left(v_{y(k)}+V_{y}^{W}\right)\Delta t\\ \theta_{v(k)}+\left(v_{\theta (k)}+V_{\theta }^{W}\right)\Delta t\\ \begin{array}{l} v_{x(k)}+V_{x}^{W}\\ v_{y(k)}+V_{y}^{W}\\ v_{\theta (k)}+V_{\theta }^{W} \end{array} \end{array}\right]$$

An Extended Kalman Filter propagates the sensor pose and velocity estimates, as well as feature estimates.

### 3.2. Features definition and measurement model

**Figure 1.**
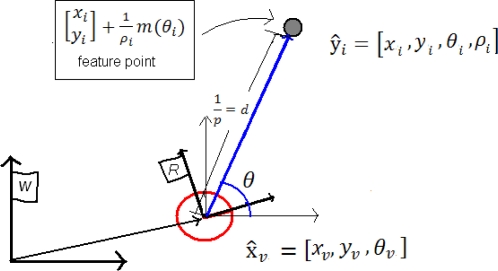
Inverse depth parameterization.

The complete state x̂ that includes the features ŷ is made of:

(4) $$\hat{x}={\left[{\hat{x}}_{v}{,\hat{y}}_{1},{\hat{y}}_{2},...{\hat{y}}_{n}\right]}^{T}$$

where a feature ŷ represents a feature *i* defined by the 4-dimension state vector:

(5) $${\hat{y}}_{i}={\left[x_{i}{,y}_{i},\theta_{i},\rho_{i}\right]}^{T}$$

which models a 2-D point located at:

(6) $$\left[\begin{array}{c} x_{i}\\ y_{i} \end{array}\right]+\frac{1}{\rho_{i}}m\left(\theta_{i}\right)$$

where *x_i_, y_i_* is the sensor center coordinates when the feature was first observed; and *θ*_i_ represents the azimuth (respect to the world reference *W*) for the directional unitary vector *m*(*θ*_i_). The point depth *d_i_* along the ray is coded by its inverse *ρ_i_* = 1*/d_i_* (Figure 1).

The use of an inverse depth parameterization for bearing-only SLAM can improve the linearity of the measurement equation even for small changes in the sensor position (corresponding to small changes in the parallax angle), this fact allows a Gaussian distribution to cover uncertainty in depth which spans a depth range from nearby to infinity. It is well known the relevance of a good uncertainty Gaussian representation in a scheme based in EKF [24].

**Figure 2.**
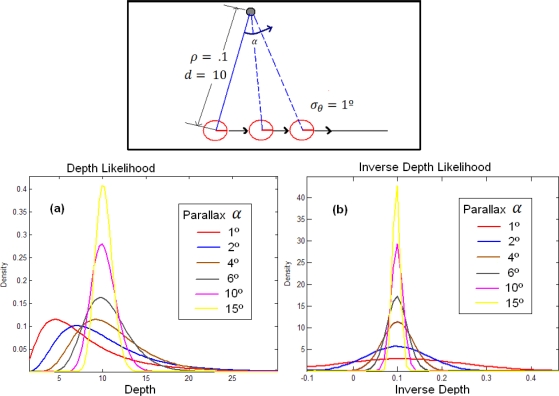
Simulation of a point reconstruction from observations with different parallax.

Figure 2 shows a simulation for a point reconstruction from noisy bearing measurements at different parallax (upper plot), using both, the Euclidean and the Inverse Depth parameterization. The location of the vehicle is known. A Gaussian error σ*_θ_* = 1° (degrees) is introduced in bearings. Lower plots show the evolution of the likelihood for depth and inverse depth as the parallax in the observation grows: in (a), the estimates of depth likelihood converge to a Gaussian-like shape, but the initial estimates are highly non-Gaussian, with heavy tails. In contrast, likelihoods of inverse depth (b) (abscissa in inverse meters) are nearly Gaussian, even for low parallax. Therefore it can be clearly appreciated how the uncertainty can be represented by a Gaussian using the inverse depth parameterization over whole parallax range, whereas the Euclidean representation converges to a Gaussian-like shape only to the final estimates. For the Euclidean representation, the parallax needed for the likelihood converges to a Gaussian-like shape, depending on the sensor noise, and thus the noisier the sensor the more parallax is needed for convergence.

The different locations of the sensor, along with the location of the already mapped features, are used to predict the feature angle *h_i_* (angle describing the direction of the feature in the sensor coordinate frame). The measurement model is defined by:

(7) $$h_{i}=\text{atan}2\left(\frac{1}{\rho_{i}}\text{sin}\;\theta_{i}+y_{i}-y_{v},\frac{1}{\rho_{i}}\text{cos}\;\theta_{i}+x_{i}-x_{v}\right)$$

atan2 is a two-argument function that computes the arctangent of *y*/*x* given *y* and *x*, within a range of [−π, π]. At this stage it is assumed that the bearing sensor is capable of tracking and discriminating between the landmarks, in other words, the data association problem is obviated.

In implementation using real data, features search could be constrained to regions around the predicted *h_i_*. These regions are defined by the innovation covariance matrix *S_i_* = *H_i_P*_*k*+1_*H′_i_* + *R* where *H_i_* is the Jacobian of the sensor model with respect to the state, *P*_*k*+1_ is the prior state covariance, and measurements *z* are assumed corrupted by zero mean Gaussian noise with covariance *R*.

As it was stated before, depth information cannot be obtained in a single measurement when bearing sensors are used. To infer the depth of a feature, the sensor must observe it repeatedly as the sensor freely moves through its environment, estimating the angle from the feature to its center. The difference between angle measurements is the feature parallax. Actually, parallax is the key that allows to estimating features depth. In the case of indoor sequences, centimeters are enough to produce parallax, on the other hand, the more distant the feature, the more the sensor has to travel to produce parallax. Therefore, in order to incorporate new features to the map, special techniques for features system-initialization are needed in order to enable the use of bearing sensors in SLAM systems.

Let us consider two methods, which represent the main approaches (undelayed and delayed) for addressing the initialization problem.

### 3.3. ID-Undelayed initialization

For the Inverse depth (ID) undelayed method presented in [22], transition from partially to fully initialized features do not need to be explicitly tackled; this means that the feature is added to the map in its final representation since the first frame was observed. The initialization includes both the feature state initial values and the covariance assignment. The initial uncertainty region covers a huge range depth [*d_min_*, ∞] as Gaussian because the low linearization errors, due to the inverse depth parameterization. Once initialized, the feature is processed with the standard EKF prediction-update loop.

Using the inverse depth parameterization, while the feature is observed at low parallax, the feature will be used mainly to determine the sensor orientation but the feature depth will be kept quite uncertain; if the sensor translation is able to produce a parallax big enough then the feature depth estimation will be improved.

For the ID-Undelayed method (Figure 1), a new feature ŷnew (Equation 5) is initialized, when is detected the first time *k*, as follows:

(8) $$\left[\begin{array}{c} x_{i}\\ y_{i}\\ \theta_{i} \end{array}\right]=\left[\begin{array}{c} x_{v}\\ y_{v}\\ \theta_{v}+z_{\theta } \end{array}\right]$$

where *x_v_*, *y_v_*, *θ_v_* are taken directly from the current state x̂*_v_* and *z_θ_* is the initial bearing measurement. The initial value for ρ*_i_* is derived heuristically to cover in its 95% acceptance region, a working space from infinity to a predefined close distance d_min_ expressed as inverse depth:

(9) $$\left[\frac{1}{d_{\text{min}}},0\right]\;\text{so}:\;\rho_{i}=\frac{\rho_{\text{min}}}{2}\;\sigma_{\rho }=\frac{\rho_{\text{min}}}{4}\;\rho_{\text{min}}=\frac{1}{d_{\text{min}}}$$

In [22] the parameters are set as *d_min_* = 1, ρ*_i_* = 0.5, σ_ρ_ = 0.25. The new system state x̂ is conformed simply adding the new feature ŷ_i_ to the final of the vector state:

(10) $$\hat{x}=\left[\begin{array}{c} {\hat{x}}_{v}\\ {\hat{y}}_{1}\\ {\hat{y}}_{2} \end{array}\right]{\;\hat{x}}_{\text{new}}=\left[\begin{array}{c} {\hat{x}}_{v}\\ {\hat{y}}_{1}\\ {\hat{y}}_{2}\\ {\hat{y}}_{i} \end{array}\right]\;$$

The state covariance after feature initialization is defined by:

(11) $$P_{\text{new}}=J\left[\begin{array}{ccc} P_{\left(k\right)} & 0 & 0\\ 0 & R_{j} & 0\\ 0 & 0 & \sigma_{\rho }^{2} \end{array}\right]J^{\prime }$$

being *J* the Jacobian for the initialization function.

### 3.4. ID-Delayed initialization

In experiments using the undelayed initialization, it often happens that the inverse depth becomes negative after a Kalman update, due to the observation noise that predominates over the update of the depth, but there are simple solutions to solve this problem. Moreover, when an initial metric reference is used in order to recover/set the scale of the map (very relevant for robotics applications), initial fixed parameters (inverse depth and uncertainty) must be tuned in order to ensure convergence.

**Figure 3.**
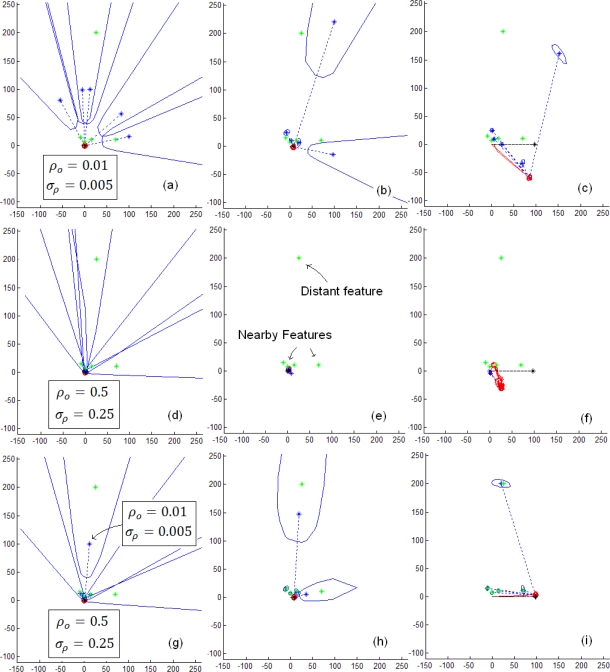
Some issues of the ID-Undelayed initialization.

Figure 3 illustrates the SLAM process using the ID-Undelayed following a simple straight trajectory for 5 features, (4 of them near to the vehicle and the other more distant). In upper plots (a,b and c) the initial parameters (*ρ_o_* = 0.01, σ*_ρ_* = 0.005) are set in order to initialize the features at the middle of the distance between the vehicle and the more distant feature, and therefore far enough respect to the nearby features (plot *a*). In this case we found in almost every case a huge drift in the estimates (plot *c*), and it can be appreciated that initializing the features far away from the sensor fails if there are some closest landmarks. If other values (*ρ_o_* = 0.5, *σ_ρ_* = 0.25) are used (near to the sensor), (central plots *d*, *e* and *f*) then the percentage of convergence is poor (approx 50%) (plot *f*), due to the influence of the distant feature. In this case if the distant feature is removed from the map (not illustrated here) then a percentage of convergence of 80% is achieved. In the last series (lower plots *g*, *h* and *i*) we combine both initial values: *ρ_o_* = 0.01, *σ_ρ_* = 0.005 for the distant landmark and *ρ_o_* = 0.5, *σ_ρ_* = 0.25 for the all the nearby ones. In this case an effectiveness of 90% was achieved for the algorithm.

The issues mentioned above suggest us that the initial inverse depth and their associated initial uncertainty of the new features added to the map could be treated before to be added to the system state instead of use fixed initial depth and uncertainty. In [23] a delayed version of an undelayed method is proposed. In this case, initial depth and uncertainty of each feature are dynamically estimated prior to adding the new landmark in the stochastic map.

For the ID-Delayed method, a new feature *ŷ_new_* (Equation 5) is initialized as follows:

When a feature is detected the first time *k*, some part of the current state x̂ and covariance matrix P together with the sensor measurement are stored, this data λ (called *candidate points*) is composed by:

(12) $$\lambda_{i}=\left[x_{1},y_{1},\theta_{1},z_{1},\sigma_{1}^{x},\sigma_{1}^{y},\sigma_{1}^{\theta }\right]$$

**Figure 4.**
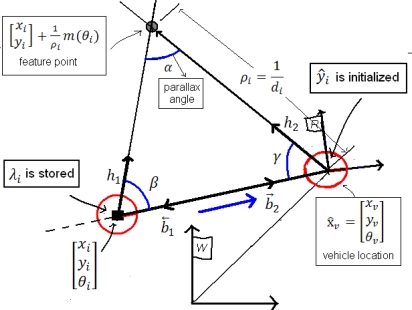
ID-Delayed parameterization.

The values *x*_1_, *y*_1_ and *θ*_1_ represent the current robot position; *σ*_1_^*x*^, *σ*_1_^*y*^ and *σ*_1_^*θ*^ represent their associated variances taken from the state covariance matrix P_k_ and z_1_ is the first bearing measurement to the landmark. In subsequent instants *k*, the feature is tracked until a minimum parallax threshold *α_min_* is reached. Figure 4 shows that a few degrees of parallax are enough to reduce the uncertainty in the estimation.

The parallax α is estimated using:

- The base-line *b*.
- *λ_i_*, using its associated data (*x*_1_, *y*_1_, *θ*_1_, *z*_1_, *σ*_1_*^x^*, *σ*_1_*^y^*, *σ*_1_*^θ^*).
- The current state (*x*_v_, *y*_v_, *θ*_v_, *z*, *σ^x^*_v_, *σ^y^*_v_, *σ^θ^*_v_).

For each candidate point λ_i_, every time that a new bearing measurement *z* is available, the parallax angle *α* can be estimated as (Figure 4):

(13) α = π− (β + γ)

The angle *β* is determined by the directional unitary vector *h*_1_ and the vector *b*_1_ defines the base-line *b* in the direction of the sensor trajectory.

The angle *γ* is determined in a similar way as *β* but using the directional unitary vector *h*_2_ and the vector *b*_2_ defining the base line in the opposite direction of the sensor trajectory by:

(14) $$\beta =\cos^{-1}\left(\frac{h_{1}\cdot b_{1}}{\left\|h_{1}\right\|\left\|b_{1}\right\|}\right)\;\gamma ={\text{cos}}^{-1}\left(\frac{h_{2}\cdot b_{2}}{\left\|h_{2}\right\|\left\|b_{2}\right\|}\right)$$

where (*h*_1_ · *b*_1_) is the dot product between *h*_1_ and *b*_1_. The directional vector *h*_1_, expressed in the absolute frame W, points from the sensor location to the direction when the landmark was observed for the first time, and is computed using the data stored in λ_i_ denoting the bearing *z_i_*. The directional vector *h*_2_ expressed in the absolute frame W is computed in a similar way as *h*_1_ but using the current sensor position *x̂_v_* and the current measurement *z_i_*.

(15) $$h_{1}=\left[\begin{array}{c} \text{cos}\;\left(\theta_{1}+z_{1}\right)\\ \text{sin}\;\left(\theta_{1}+z_{1}\right) \end{array}\right]\;h_{2}=\left[\begin{array}{c} \text{cos}\;\left(\theta_{v}+z_{i}\right)\\ \text{sin}\;\left(\theta_{v}+z_{i}\right) \end{array}\right]$$

*b*_1_ is the vector representing the robot base-line between the robot center position *x*_1_, *y*_1_ stored in *λ_i_* where the point was first detected and the current sensor center (*x*_v_, *y*_v_). *b*_2_ is equal to *b*_1_ but pointing to the opposite direction. The base-line *b* is the module of *b*_2_ or *b*_1_:

(16) $$b=\left\|b_{1}\right\|=\left\|b_{2}\right\|\;b_{1}=\left[\left(x_{v}-x_{1}\right),\left(y_{v}-y_{1}\right)\right]\;b_{2}=\left[\left(x_{1}-x_{v}\right),\left(y_{1}-y_{v}\right)\right]$$

If α > α_min_ then *λ_i_* is initialized as a new feature ŷ_i_. The threshold α_min_ can be established depending on the accuracy of the bearing sensor. Depth uncertainty is reasonably well minimized when *α* = 10°.

For a new feature ŷ_i,_ values of *x_i_*, *y_i_*, *θ_i_* are defined in the same way as Equation 8. For the delayed approach the dynamical estimation of ρ*_i_* is derived from:

(17) $$\rho_{i}=\frac{\text{sin}\;\alpha }{b\;\text{sin}\;\beta }$$

The variance *σ_ρ_* for the inverse depth *ρ* is calculated now from the initialization process, instead of a variance predefined heuristically as it was made in the undelayed method, therefore the covariance for the new feature ŷ_new_ is derived from the error diagonal covariance matrix *R_i_* measurement and the state covariance matrix P.

(18) $$R_{i}=\mathrm{diag}\left[\sigma_{1}^{x},\sigma_{1}^{y},\sigma_{1}^{\theta },\sigma_{z}^{2},\sigma_{z}^{2}\right]$$

For reasons of simplicity *R_i_* is defined as a diagonal matrix (cross-covariances are not taken into account) and is now conformed by the error variance of the standard deviation of the bearing sensor *σ_z_* (one for each bearing estimation *z*_1_ and *z*) and the variances stored in *λ_i_* (*σ*_1_*^x^*, *σ*_1_*^y^* and *σ*_1_*^θ^*). Note that the value of σ_z_ is constant and is not stored previously in Equation 12.

The new state covariance matrix, after initialization, is:

(19) $$P_{\text{new}}=J\left[\begin{array}{cc} P_{k} & 0\\ 0 & R_{j} \end{array}\right]J^{\prime }$$

Note that unlike the ID-Undelayed method there is not an implicit initial uncertainty in depth σ_ρ_ (Equation 11). In the ID-Delayed method the complete covariance for the new feature is fully estimated by the initialization process.

### 3.5. Undelayed *vs.* Delayed

In an Undelayed approach, when a feature is added to the map the first time that it has been observed, its depth is modeled with a huge uncertainty. In that sense, this new feature does not provide any depth information. However, at this stage the benefit of the Undelayed approach is that features provide information about the sensor orientation from the beginning.

On the other hand, it can be useful to wait until the sensor movement produces some degrees of parallax, (gathering depth information) in order to improve robustness, especially when an initial metric reference is used for recovering scale. Moreover, when cameras are used in real cluttered environments, the delay can be used for efficiently reject weak features, thus initializing only the best features as new landmarks to the map.

## 4. Concurrent Initialization

**Figure 5.**
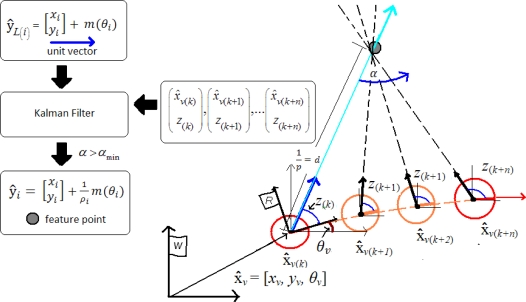
Concurrent initialization process.

This section presents a novel and robust method, called *Concurrent Initialization,* for initializing new features in bearing-sensor-based SLAM systems. The method takes advantage of both, undelayed and delayed approaches. When a feature is detected for the first time, it is immediately initialized in the map (undelayed) as a directional vector which contributes since the beginning to the estimation of the sensor orientation. After that, while the sensor moves freely through its environment, the incoming measurements are incorporated via an uncorrelated linear Kalman filter in order to estimate the depth of the feature. If the sensor movement produces enough parallax, then the feature will be updated with the estimated depth, and thus also contributing to the estimation of the sensor location. Very far features will not produce parallax, and will remain in the form of a directional vector in the map, but contributing to the estimation of sensor orientation. Figure 5 illustrates the concurrent initialization process.

### 4.1. Undelayed stage

When a feature is detected for the first time *k*, it is initialized immediately in the map as a new landmark ŷ_L(i)_ which is composed by the 3-dimension state vector:

(20) $${\hat{y}}_{L(i)}={\left[x_{i}{,y}_{i},\theta_{i}\right]}^{T}$$

where

(21) $$\left[\begin{array}{c} x_{i}\\ y_{i}\\ \theta_{i} \end{array}\right]=\left[\begin{array}{c} x_{v}\\ y_{v}\\ \theta_{v}+z_{\theta } \end{array}\right]$$

*x_v_*, *y_v_*, *θ_v_* are taken directly from the current state x̂*_v_* and *z_θ_* is the initial bearing measurement. ŷ_L(i)_ defines a directional vector, expressed in the absolute frame W, which represents the direction of the landmark from the sensor, when it was observed for the first time. The covariance matrix P is updated in the same manner as Equation 19 but using the proper Jacobian *J*.

Parallel to the system state (represented by the state vector x̂), a state vector x̂_can_ is used for estimating (via an extra linear Kalman Filter) the feature depth of each landmark ŷ_L_. The state x̂_can_ is not directly correlated with the map.

Every time a new feature ŷ_L(i)_ is initialized in x̂, the state x̂_can_ is augmented as:

(22) $${\hat{x}}_{\text{can}}=\left[\begin{array}{c} \lambda_{1}\\ \lambda_{2} \end{array}\right]{\;\hat{x}}_{\text{can}\_\text{new}}=\left[\begin{array}{c} \lambda_{1}\\ \lambda_{2}\\ \lambda_{i} \end{array}\right]\;$$

where λ_i_ is a 3-dimension vector composed by:

(23) $$\lambda_{i}=\left[\alpha_{i},\Delta \alpha_{i},\rho_{i}\right]\prime$$

For each λ_i_, α_i_ is the estimated parallax, Δα_i_ is the rate of change in parallax and ρ*_i_* is the estimated inverse depth.

The covariance matrix of x̂_can_, P_can_, is augmented simply by:

(24) $$P_{\text{can}\_\text{new}}=\left[\begin{array}{cc} P_{\text{can}} & 0\\ 0 & R_{c} \end{array}\right]$$

The three initial values of λ_i_ are set to zero, and the initial values of R_c_ have been heuristically determined as: R_c_ = *diag*(.01, .01, 1).

### 4.2. Delayed stage

While the sensor moves through its environment, it can observe repeatedly a landmark ŷ_L(i)_, at each iteration generating a new angle measurement *z*. All these new measurements are successively added to the linear Kalman Filter (responsible for estimating x̂_can_) in order to infer the landmark depth. For each new measurement *z_i_* of a feature ŷ_L(i)_ an iteration of the filter is executed.

The state transition model for each λ_i_ is:

(25) $${\;\hat{x}}_{\text{can}\_i(k+1)}=\left[\begin{array}{c} \alpha_{i(k+1)}\\ \Delta \alpha_{i(k+1)}\\ \rho_{i(k+1)} \end{array}\right]=\left[\begin{array}{c} \alpha_{i(k)}+\Delta \alpha_{i(k)}\\ \Delta \alpha_{i(k)}\\ \rho_{i(k)} \end{array}\right]$$

A process noise w*_k_* ∼ *N*(0,Q*_k_*) is considered. In experiments: Q*_k_* = *diag*(8e^−7^, 10e^−9^) have been used.

The measurement prediction model is directly obtained from the state. On the other hand, the measurements *z_can_* used to update the filter are a function of: (i) the feature ŷ_L(i)_, (ii) the sensor state x̂*_v_* and (iii) the current measurement *z_i_*.

(26) $$\;z_{\mathrm{can}}=\left[\begin{array}{c} z_{\alpha }\\ z_{\rho } \end{array}\right]=f_{z}\left({\hat{y}}_{L(i)},{\hat{x}}_{v},z_{i}\right)$$

*z_α_* and *z_ρ_* are estimated in the same manner as Equation 13 and Equation 17 respectively. Only note that in Equation 16, *x*_1_ and *y*_1_ are taken from ŷ_L(i)_, so *x*_1_ = *x_i_* and *y*_1_ = *y_i_*.

The implicit uncertainties in the estimation of the function *f_z_* are used to compose the error measurement covariance matrix R_can_:

(27) $$R_{\mathrm{can}}={\nabla f}_{z}(P_{t}){\nabla f}_{z}\prime$$

where Δ*f_z_* is the Jacobian of *f_z_* with respect to *z*_can_. P_t_ is formed by:

(28) $$P_{t}=\left[\begin{array}{ccc} P_{{\hat{x}}_{v}} & P_{{\hat{x}}_{v}{\hat{y}}_{L(i)}} & 0\\ P_{{\hat{y}}_{L\left(i\right)}{\hat{x}}_{v}} & P_{{\hat{y}}_{L(i)}} & 0\\ 0 & 0 & \sigma_{z}^{2} \end{array}\right]$$

All the components of P_t_, except σ_z_ (the error variance of the bearing sensor) are taken directly from the covariance matrix P of the system state x̂. P_x̂*v*_ is the submatrix of P corresponding to the covariance of the sensor state x̂_v_. P_ŷ_L(i)__ is the covariance of the feature ŷ_L(i)_. P_x̂_v_ŷ_L(i)__ and P_ŷ_L(i)_x̂_v__ are the correlations between x̂_v_ and ŷ_L(i)_.

R_can_ is used in the Kalman update equations for estimating the innovation covariance matrix *S_i_*.

### 4.3. Updating depth

Features expressed in the form of ŷ_L(i)_ are very useful to estimate the sensor orientation *θ_v_*. In [25] a visual compass is proposed based in this fact. Besides, the depth of features are needed for estimating the sensor location [*x_v_*,*y_v_*]. For near features ŷ_L(i)_, a small sensor translation is enough to produce some parallax and thus to infer depth.

**Figure 6.**
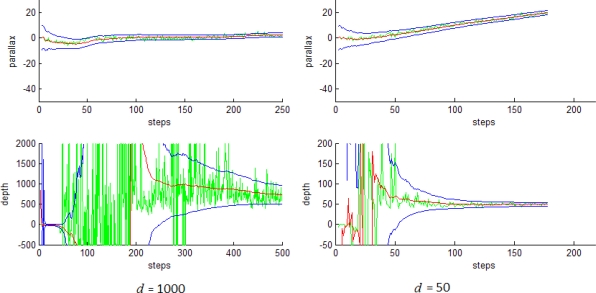
Parallax and depth estimations for a distant and a close feature.

The state x̂_can_ encloses the parallax α_i_ and inverse depth ρ*_i_* estimations for each feature ŷ_L(i)_. Figure 6 shows the evolution of parameters α_i_ (upper plot) and *d* = 1/ρ*_i_* (lower plot) and its uncertainty for the feature estimated by the linear Kalman filter, (left plot) feature at a distance of *d* = 1,000 units and (right plot) for a distance of *d* = 50 units. The boundary uncertainties at 3σ are indicated in blue color. The filtered values are depicted in red color. Also note in green color the raw measurements *z*_can_ (taken from Equation 26). In these graphics it can be clearly appreciated how the estimation of depth *d* is directly influenced by the parallax; for the near feature, about 100 steps are needed to producing parallax and thus *d* converges rapidly to its real value. Also note that the uncertainty is rapidly minimized. On the other hand, the distant feature produces small parallax. For a low parallax the sensor noise predominates and therefore produces very fluctuant raw measurements. Even so, after several steps, the filter estimates the depth reasonable well with a high related uncertainty.

A minimum parallax threshold α_min_ is used for updating a feature ŷ_L(i)_ as ŷ_i_. Distant features will not produce parallax and therefore will remain expressed as ŷ_L(i)_, but contributing to the estimation of sensor orientation *θ_v_*.

On the other hand, if α_i_ > α_min_ then:

(29) $${\hat{y}}_{L(i)}={\left[x_{i},y_{i},\theta_{i}\right]}^{T}\to {\hat{y}}_{i}={\left[x_{i},y_{i},\theta_{i},\rho_{i}\right]}^{T}$$

where *ρ_i_* is taken directly from x̂_can_. The covariance matrix P is transformed by the corresponding Jacobian:

(30) $$P_{\text{new}}=J\left[\begin{array}{cc} P & 0\\ 0 & {q\sigma }_{\rho_{i}}^{2} \end{array}\right]J\prime$$

being σ_ρ_^y^ the variance of the inverse depth estimation for ŷ_L(i)_ and taken from P_can_. The constant *q* is used to increase the initial uncertainty of ρ*_i_* in order to improve filter consistency. In experiments *q* is set to 100.

(31) $$J=\left[\begin{array}{cccc} I & 0 & 0 & 0\\ 0 & \frac{\partial {\hat{y}}_{i}}{{\partial \hat{y}}_{L(i)}} & 0 & 1\\ 0 & 0 & I & 0 \end{array}\right]$$

When a feature ŷ_L(i)_ is updated as ŷ_i_, then its corresponding values will be removed from the linear Kalman filter responsible for estimating the state x̂_can_.

### 4.4. Measurement

At any time, the map can include both kind of features ŷ_L(i)_ and ŷ_i_. Thus each kind of feature has its own measurement prediction model:

- For features ŷ_i_ measurement Equation 7 is used. A value of 2 to 6 times the real error of the bearing sensor is considered for the measurement process.
- Features ŷ_L(i)_ are supposed to be very far from the sensor and therefore it is assumed that its corresponding bearing measurement *z_i_* will remain almost constant. The measurement prediction model is simply:
  (32) $$h_{L(i)}=\theta_{i}-\theta_{v}$$
- For near features ŷ_L(i)_, the bearing measurement *z_i_* will change rapidly. Due to this fact the standard deviation of the bearing sensor σ_z_ for features ŷ_L(i),_ (in update Kalman equations) is multiplied by a high value *c*, (*c* = 10e^10^ were used in experiments). An interesting issue, to be treated for further work, could be the dynamical estimation of parameter *c*.

Both kinds of measurement prediction models must be used together, if several measurements *z_i_* are made at the same time for both kinds of features. For example, consider that the bearing sensor takes measurements of the features ŷ_L(2)_ and ŷ_3_ simultaneously, then:

(33) $$v=\left[\begin{array}{c} z_{2}\\ z_{3} \end{array}\right]-\left[\begin{array}{c} h_{L(2)}\\ h_{3} \end{array}\right]\;R=\left[\begin{array}{cc} {c\sigma }_{z}^{2} & 0\\ 0 & \sigma_{z}^{2} \end{array}\right]$$

(34) $$\nabla H=\left[\begin{array}{ccccc} \frac{{\partial h}_{L(2)}}{{\partial \hat{x}}_{v}} & 0 & \frac{{\partial h}_{L(2)}}{{\partial \hat{y}}_{L(2)}} & 0 & 0\\ \frac{{\partial h}_{3}}{{\partial \hat{x}}_{v}} & 0 & 0 & \frac{\partial h_{3}}{{\partial \hat{y}}_{3}} & 0 \end{array}\right]$$

where *v* is the innovation, R is the error covariance matrix and ∇H is the Jacobian measurement model.

## 5. Experiments

Several simulations have been executed in order test the performance of the proposed method in relation to other approaches. Simulations are extremely helpful when different methods are compared among others, because numerous variables (inherent to real environments) are obviated (e.g., data association), or become constants (*i.e.*, synthetic map), therefore the cores of the methods are compared.

### 5.1. Initialization process of distant and near features

**Figure 7.**
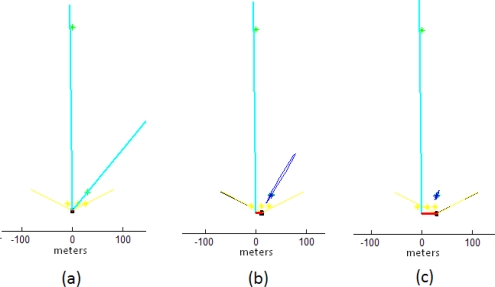
Sequence illustrating the concurrent initialization process: (a) beginning of initialization; (b) initialization process at step 100; (c) initialization process at step 250.

Figure 7 shows the evolution in the initialization process of two features maps with the concurrent initialization: a distant one (600 units of depth) and a near one (50 units of depth). The only information given a priori to the system was the scale reference (the three points in yellow) which was introduced with an associated uncertainty close to zero in the covariance matrix R. Taking into account that there is not an additional sensory input (e.g., odometry), at every step an unknown linear and angular acceleration is introduced with zero mean and known-covariance Gaussian processes (Section 3.1). In this case, *a*^W^_x_ = 4 m/s^2^, *a*^W^_y_ = 4 m/s^2^ and *a*^W^_θ_ = 2 rad/s^2^ were used. The only input sensor of the system is a noisy sensor capable of measuring the bearing of features, with a limited field of view of 110° (emulating a 2-DOF camera). A standard deviation of σ = 1° is considered for the sensor readings.

At the begin of the sequence (plot *a*) both features has been initialized as a directional vector ŷ_L(i)_, defining a ray (in cyan color). Note that three feature points (in yellow color) have been previously added to the map as a priori known landmarks in order to define/set the scale of the world. Around step 100 (plot *b*), the small displacement of the sensor to the right produces enough parallax to estimating the depth of the near feature. Observe that the near feature ŷ_L(i)_ has been transformed to a ŷ_i_ feature (blue color). Also note that some uncertainty (especially in depth) remains at the moment of the transformation. By the last step, at 250 iterations, (plot *c*) the uncertainty in the near feature has been full minimized, on the other hand, the movement of the sensor has not produced enough parallax and therefore, the distant feature remains in the form of ŷ_L(i)_ but still contributing to the estimation of the sensor orientation.

### 5.2. Comparative study

In order to show the performance of the Concurrent method proposed in this article, a comparative study between the ID-Undelayed method [22] and the ID-Delayed [23] method is presented.

**Figure 8.**
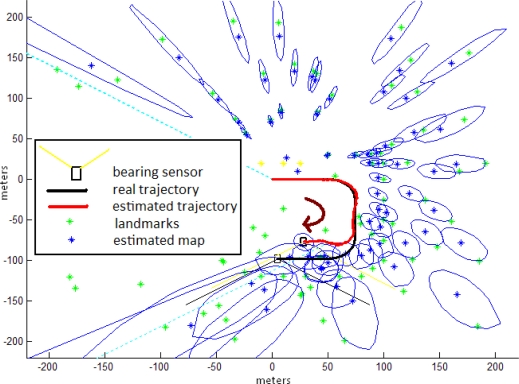
SLAM simulation using the concurrent initialization method.

Figure 8 illustrates the environment setup used in the study. For all the tests, the bearing sensor is moved over a semi-cycled U-like shape trajectory, since our main goal is to observe the effect of the initialization process of new features in the estimation of both map and sensor location, instead of the closing loop problem. About 100 landmarks (in green) simulate the environment of the sensor. Figure 8 also shows both the map and sensor trajectory estimates after a single run of 2,000 steps of the Concurrent method. The features map and their uncertainties are indicated in blue. Note the evolution of uncertainty in both sensor and features location. Also note the typical (in SLAM systems) drift in both trajectory and map estimations as the sensor moves far away from its initial location.

The *average* NEES (normalised estimation error squared [26]) over N Monte Carlo runs of the filter was used in order to evaluate the consistency of the methods, as it is proposed in [27]. The NEES is estimated as follows:

(35) $$\varepsilon_{k}={\left[x_{k}-{\hat{x}}_{k|k}\right]}^{T}P_{k|k}^{-1}\left[x_{k}-{\hat{x}}_{k|k}\right]$$

where x*_k_* is the true state and the average NEES is computed as:

(36) $${\bar{\varepsilon }}_{k}=\frac{1}{N}\sum_{i=1}^{N}\varepsilon_{k(i)}$$

Four different tests were realized to comparing the methods under diverse conditions. Table 2 shows the values for the linear and angular acceleration (*a*^W^_x_, *a*^W^_y_ and *a*^W^_θ_ in 4m/s^2^) and the time between “frames” (Δ*t* in seconds) used for each test. In this case, a higher Δ*t* implies bigger displacements of the robot from frame to frame and therefore more linearization errors due to large changes in parallax.

In experiments *ρ_ini_* = 0.05 and *σ_ρ_* = 0.025 were used for the ID-Undelayed method and α_min_ = 10° were used for both ID-Delayed and Concurrent methods. The NEES was estimated over the 3-dimensional robot pose. The average NEES for each method was estimated using N=20 Monte Carlo runs. In simulations the sensor was moved 3 meters every Δ*t* = 1 second for the straight sections and 4.5 meters every Δ*t* = 1 seconds for the curve sections of the trajectory.

MATLAB code was run using a 1.73 GHz Pentium M laptop. Table 3 shows the execution time of the three methods for conditions Δ*t* = 1/30 and Δ*t* = 1/120. As it would be expected, for Δ*t* = 1/120 the execution time was around four times longer than Δ*t* = 1/30, for the same trajectory. Execution time of the Concurrent method was estimated using a diagnostic version of the algorithm, marked with an asterisk (*) in Table 3. This version uses a 5*n* Kalman filter (instead of 3*n*) which estimates two extra diagnostic parameters. This means that the real execution time of the Concurrent method should be somewhat faster.

For some runs of the algorithms, filter divergence could occur. Table 3 also shows the failed attempts, this means the number of times that filter divergence appeared before a method reach N = 20 positives runs (with convergence), for each test. The average NEES was only estimated using positive runs.

**Figure 9.**
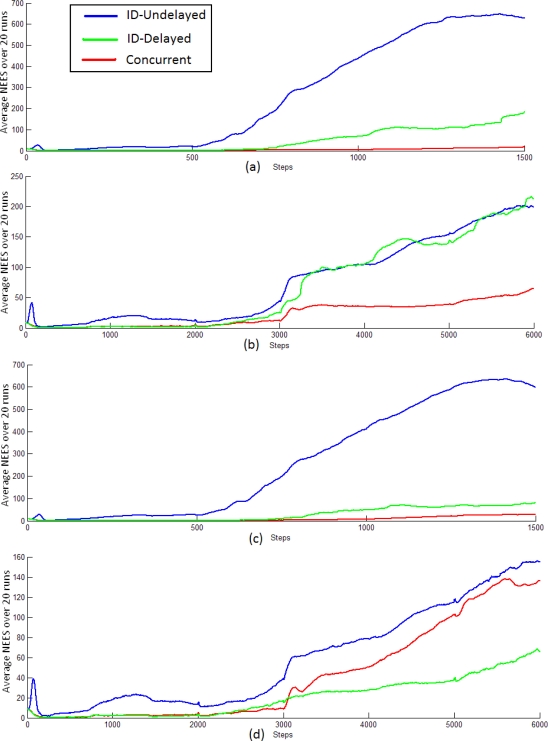
Comparison of the average NEES for ID-Undelayed, ID-Delayed and Concurrent methods.

Figure 9 shows the evolution of the average NEES (*ε̄_k_*) for tests (a), (b), (c), (d) for each method. Note that test (b) and (d) need four times more steps than test (a) and (c) in order to complete the same trajectory due to their particular value of Δ*t*.

As it would be expected, the whole methods become optimistic after a certain time [27]. Nevertheless, it can be observed for tests (a), (b) and (c) that *ε̄_k_* achieved for Concurrent method (red) tends to be lower than *ε̄ _k_* achieved for both ID-Undelayed (blue) and ID-Delayed (green) methods. This behavior is more evident in test (a) and (c), where lower noise is injected into the sensor motion model. The difference between (a) and (c) is the time between “frames” (Δ*t*) used for each test. The above results suggest, that if adequate noise is injected, then Concurrent method seems to be less sensitive to large jumps in parallax (and linearization errors), and therefore could be suitable for application where a high “frame-rate” is not available. Regarding to the ID-Undelayed method, it can be observed the effect of the parameter Δ*t* over the magnitude of *ε̄_k_*, although its form is somewhat similar for all tests. In test (a) and (c) where Δ*t* is higher (1/30), *ε̄_k_* reaches around 600 units, while in test (b) and (d) where Δ*t* is lower (1/120) *ε̄_k_* reaches around 200 units. Theoretically the *ε̄_k_* for the ID-Undelayed method would be more favorable as Δ*t*→0. At this point, it is important to remember that implementation of Concurrent method implies the estimation of an extra linear Kalman filter of dimension 3*n*, where *n* is the number of features ŷ_L(i)_ in the state x̂. On the other hand, the Concurrent method is lees sensitive to the parameter Δ*t*. At least for this experimental setup, the average NEES for the Concurrent method, when Δ*t* = 1/30 (94 s of execution time), is even lower than the *ε̄_k_* for the ID-Undelayed, when Δ*t* = 1/120 (302 s of execution time). The ID-Delayed method represents the most efficient alternative in computational cost with medium performance in average NEES terms. However the ID-Delayed method shows to be the least robust method in terms of convergence (Table 3). The problem of convergence for the ID-Delayed method is notorious in tests (a) and (c) where Δ*t* is higher (1/30), these results indicate a frame-rate dependency of the method. On the other hand, for the realized tests, the Concurrent method shows the best results in terms of convergence.

## 6. Conclusions

This work proposes a novel and robust approach for initializing new features in SLAM systems based in bearing sensors. First, an overview of the problem is given and the most relevant related work is presented. Most of the methods presented in the literature can be classified into two categories: Undelayed and Delayed Methods. In that sense, an analysis of two representative methods of the aforementioned taxonomy is also presented. Undelayed methods provide information of orientation since a feature is first detected, on the other hand, Delayed methods await until some depth information is gathered, improving convergence.

The proposed approach in this article, the concurrent initialization method, takes the best of both, Undelayed and Delayed approaches. The key is to use two kinds of feature representations concurrently for both undelayed and delayed stages of the estimation. The simulations results, based in the average NEES test, showed that the Concurrent method can maintain the filter consistency satisfactorily. Moreover, observing the percentage of convergence, the Concurrent method appears also to be robust.

It is important to mention that the complexity of the Concurrent method is higher than other methods (*i.e.*, ID-Undelayed method) and the additional Kalman filter certainly implies an increase of computational requirements per frame. On the other hand, the Concurrent method appears to be less sensitive to the linearization errors induced for large jumps in parallax (much time between frames). In that sense the Concurrent method could be even more efficient in computational terms than other methods because it seems to work properly at low frame rate. This attribute also makes it suitable for applications where a high frame rate is not available for different reasons. The concurrent initialization method could be a robust alternative to bearing sensor based SLAM systems.

## Figures and Tables

**Table 1.** Summary of methods.

**Approach**	**Delayed / Undelayed**	**Initial representation**	**Estimation**
Deans [9]	Delayed	Bundle adjustment	EKF
Strelow [10]	Delayed	Triangulation	IEKF
Bailey [11]	Delayed	Triangulation	EKF
Davison [16]	Delayed	Multi-Hypotheses	EKF
Kwok (a) [13]	Delayed	Multi-Hypotheses	PF
Kwok (b) [14]	Undelayed	Multi-Hypotheses	PF
Sola [18]	Undelayed	Multi-Hypotheses	EKF
Lemaire [19]	Delayed	Multi-Hypotheses	EKF
Jensfelt [17]	Delayed	Triangulation	EKF
Eade [20]	Delayed	Single Hypotheses	FastSLAM
Montiel [22]	Undelayed	Single Hypotheses	EKF
Munguía[23]	Delayed	Triang.- S. Hypotheses	EKF
**This work**	Delayed-Undelayed	KF-Triang.- S.	EKF

**Table 2.** Setup of the tests.

**Test**	***a*^W^_x_**	***a*^W^_y_**	***a*^W^_θ_**	**Δ*t***
**a**	4	4	2	1/30
**b**	4	4	2	1/120
**c**	6	6	3	1/30
**d**	6	6	3	1/120

**Table 3.** Execution time & Failed attempts.

**Method**	Δ*t* = 1/30	Δ*t* = 1/120	**a**	**b**	**c**	**d**
ID-Undelayed	76s	302s	2	1	0	2
ID-Delayed	60s	235s	9	2	11	4
Concurrent	94s*	376s*	0	0	0	0
